# Supplementary material for: Stacking Model‐Based Classifiers for Dealing With Multiple Sets of Noisy Labels
Source: Biom J. 2025 Mar 12;67(2):e70042. doi: 10.1002/bimj.70042 (PMC11898607; doi:10.1002/bimj.70042)
Supplement: Supplementary file 1 — Supporting Information [file BIMJ-67-e70042-s001.zip › density-based-ensemble-model-main/competitors_real_data/netlab-master/netlab-master/help/mdnerr.htm]

Netlab Reference Manual mdnerr


# mdnerr

## Purpose

Evaluate error function for Mixture Density Network.

## Synopsis

```
e = mdnerr(net, x, t)
```

## Description

`e = mdnerr(net, x, t)` takes a mixture density network data
structure `net`, a matrix `x` of input vectors and a matrix
`t` of target vectors, and evaluates the error function
`e`. The error function is the negative log likelihood of the
target data under the conditional density given by the mixture model
parameterised by the MLP. Each row of `x` corresponds to one
input vector and each row of `t` corresponds to one target vector.

## See Also

`mdn`, `mdnfwd`, `mdngrad`

---

**Pages:**
Index


---

Copyright (c) Ian T Nabney (1996-9)

David J Evans (1998)
